# Michigan Ways

**Published:** 1900-08

**Authors:** 


### MICHIGAN WAYS.

MICROSCOPES seem to tell varying tales according to the eye that peers through the lens. Anthrax and braxy, in sheep, each finds its advocates and devotees in the reports of the recent losses occuring in this State.

The pathway of veterinary legislation in Michigan was not strewn with roses, but was rather a bed of thorns.

President Jopling, of the Michigan State Association, was active in the rôle of secretary for several years, and only illness in his immediate family has prevented more aggressive work on his part in the higher office of president.

Governor Pingree does not seem to be in accord with a large number of the profession in Michigan.

Dean Conkey, of the Grand Rapids Veterinary College, is ever ready to defend this college against all comers.

From Michigan came strong protests against our former army-bill, that denied eligibility to army veterinary service of graduates of foreign schools.

The presence in the "Wolverine State" of so many graduates of Canadian colleges seems to have surrounded the settlement of many professional questions with added difficulties.

As there seems to be many of the profession in the State in favor of a three-years college clause in fixing the status of professional requirements for veterinary practice, and the Grand Rapids College claims to have demanded the same, there would seem no good reason for not amending the law in that direction at the next session of the State legislature.

Dr. W. W. Thorburn, of the Michigan State Veterinary Board, is a graduate of the Ontario Veterinary College, class of 1883.

The exactions of the entrance requirements of the Veterinary Department of the Detroit College of Medicine were such as to save it from the responsibility and parentage of unprepared graduates of veterinary medicine, and will add greatly to the honor of those who directed its affairs.

